# Assessment of Anxiety Associated With MRI Examination Among the General Population in the Western Region of Saudi Arabia

**DOI:** 10.7759/cureus.34531

**Published:** 2023-02-02

**Authors:** Nasser M Al Shanbari, Sultan F Alobaidi, Roudin Alhasawi, Amjad S Alzahrani, Bassam M Bin Laswad, Abdulkarem A Alzahrani, Lujain F Alhashmi Alamer, Turki Alhazmi

**Affiliations:** 1 Department of Medicine and Surgery, College of Medicine, Umm Al-Qura University, Makkah, SAU; 2 Department of Medical Imaging, College of Medicine, Umm Al-Qura University, Makkah, SAU

**Keywords:** saudi arabia, patient experience, claustrophobia, patient care, mri examinations, radiology, magnetic resonance imaging, anxiety

## Abstract

Background

While magnetic resonance imaging (MRI) is one of the most efficient diagnostic methods used today, some patients may find an MRI examination to be a frightening experience. The proximity to the machine during screening and being in a confined space can cause a feeling of claustrophobia. Severe anxiety during MRI screening can cause the patient to move, which lowers the quality of the imaging and diagnostic test, and can result in the early termination of the MRI examination and the patient declining further testing.

Objectives

The objective of this study is to evaluate MRI examination-associated anxiety among Saudi Arabia’s general population in the western region of the country.

Methods

Altogether, 465 participants who had undergone an MRI examination in the western region of Saudi Arabia were recruited for this cross-sectional study. We used the Magnetic Resonance Imaging-Anxiety Questionnaire (MRI-AQ) to collect data.

Results

Regarding anxiety symptoms, 82.8% of the participants believed that they had control over the event, 80.2% were concerned beforehand, 74% required more specific information, just 48% had difficulty breathing, and 51% were panicked. On the other hand, 57.4% felt safe, 56.8% were calm, and 49.2% were relaxed. The majority of the participants (55.9%, 260) reported moderate MRI-related anxiety.

Conclusion

More than half of our respondents had mild to moderate MRI-related anxiety. The majority needed more detailed information, panicked, and had breathing problems. Statistically, females showed a significantly higher level of anxiety compared with male participants.

## Introduction

One of the most effective diagnostic techniques available is magnetic resonance imaging (MRI) [1]. This method of imaging, which uses magnetic and electromagnetic fields to cause hydrogen atoms to resonate to visualize the body’s anatomy [2], has become essential for enhancing patient care in many therapeutic disciplines [1].

It is extensively used worldwide, and its usage is growing [3]. For example, 136 and 118 MRI examinations are performed annually in Germany and the United States per 1,000 residents, respectively [2]. However, undergoing a procedure in the MRI tunnel can be an anxious experience for patients [3]. Factors such as the proximity to the machine during screening and being enclosed in a confined space can lead to a loss of control. They can also result in a sense of claustrophobia and a fear of injury, pain, and the unknown. Finally, concern about the results can also increase anxiety. Severe anxiety during MRI scanning can lead to patients moving, resulting in reduced imaging and diagnostic test quality [4]. A recent study showed that during MRI examination, male participants reported significantly greater levels of anxiety than female patients [4]. Anxiety can also lead to the premature termination of an MRI examination and patient refusal to undergo follow-up procedures [3].

Of the above, one of the most significant obstacles to effective MRI scanning is the feeling of claustrophobia, that is, the apprehension about restricted spaces. Claustrophobic patients can feel terrified and experience sensations of suffocation and repression in restricted spaces such as an MRI scanner [5]. Indeed, the feeling of claustrophobia has been shown to lead to 0.76% fewer complete examinations [6]. Another major stressor is patients’ uncertainty about their diagnosis, course of treatment, and prognosis [7].

Communication and other interpersonal interactions are relatively simple approaches to improve patient engagement and education, and may increase patients’ motivation and ability to cooperate with the MRI scanning process, as well as their perceptions of the radiology experience. Therefore, changing the way radiologists, nurses, technologists, and other members of the radiology team interact with patients before and during their MRI scan can reduce patients’ anxiety associated with MRI scanning [8].

Since claustrophobia and anxiety associated with MRI have significant impacts on the outcomes of MRI examination and patient experience, our study aims to assess the anxiety associated with MRI examination among the general population in the western region of Saudi Arabia.

## Materials and methods

Study design and participants

This cross-sectional study included as participants the general population in the western region of Saudi Arabia who had undergone an MRI examination. Participants were selected based on certain inclusion criteria, such as living in the western region. They included male and female patients, as well as Saudis and non-Saudis; all participants were above 18 years of age. We excluded participants who had never undergone an MRI examination before and who refused to participate in the study.

Ethical considerations and sample size

A self-administrated electronic questionnaire was distributed in November 2022 via social media platforms to collect data after obtaining ethical approval from the Biomedical Ethics Committee of the College of Medicine at Umm Al-Qura University, Makkah, Saudi Arabia (approval number: HAPO-02-K-012-2022-11-1233). OpenEpi version 3.0 was used to calculate the sample size of this study, keeping the confidence interval at 95% and considering a 50% prevalence of the sample size [9]. The minimally recommended calculated sample size was 385 participants. However, we included more than 450 participants to enhance the generalizability and accuracy of the results.

Study tool

This study adapted and translated a validated assessment tool [10]. After a group of expert researchers translated the questionnaire into Arabic, the face validity and content validity of the questionnaire were assessed by a certified medical imaging consultant, and the necessary modifications were undertaken. Furthermore, a pilot study was conducted to guarantee clarity and simplicity. The pilot study data were excluded from the final study results.

The study questionnaire was composed of two parts. The first part collected data on the participants’ sociodemographic characteristics such as age, sex, residency, and previous MRI examination experience. The second part consisted of the Magnetic Resonance Imaging-Anxiety Questionnaire (MRI-AQ), a 15-item questionnaire. A 4-point Likert score was used to assess the participants’ anxiety during the MRI examination. Before starting the questionnaire, consent was obtained from all participants. The contact information of the principal investigator was attached to the survey for any inquiries. All responses and data were treated with confidentiality and used for research purposes only.

Statistical analysis

After data had been extracted, they were revised, coded, and fed into the statistical software Statistical Package for the Social Sciences (SPSS) version 22 (IBM SPSS Statistics, Armonk, NY, USA). Statistical analysis was carried out using two-tailed tests. A P value of less than 0.05 was considered statistically significant. For the MRI-AQ, the total score was obtained by summing all items’ scores after reversing the scores of the relaxation items. The total score was then categorized into low anxiety (score of 15-29), intermediate anxiety (score of 30-44), and high anxiety (score of 45-60) based on the mean ± standard deviation (SD). A descriptive analysis based on the frequency and percentage distribution was carried out for all variables, including participants’ age and sex. Patients’ MRI-associated anxiety items were also tabulated, and the overall anxiety level was graphed. Participants’ mean and SD were also calculated. Cross-tabulation was used to assess the relation between MRI-associated anxiety and participants’ age and sex. These relations were tested using Pearson’s chi-square test and Fisher’s exact probability test for small frequency distributions.

## Results

A total of 465 participants who met the inclusion criteria completed the study questionnaire. The participants’ ages ranged from 18 to 70 years with a mean (SD) age of 33.4 (13.5) years; 316 (68%) were women (Table 1).

**Table 1:** Personal data of the study population who underwent MRI in the western region of Saudi Arabia MRI: magnetic resonance imaging

Personal data	Number	%
Age in years		
18-20	72	15.5%
21-30	177	38.1%
31-40	72	15.5%
>40	144	31%
Gender		
Male	149	32%
Female	316	68%

As for anxiety symptoms, 82.8% of the participants felt that they controlled the situation, 80.2% were worried in advance, 79.6% had to force themselves to manage the situation, 74% needed more detailed information, 72.7% were afraid, and 72.5% required self-control during the examination. Moreover, 48% found it hard to breathe and 51% panicked. On the other hand, 57.4% felt safe, 56.8% felt calm, and 49.2% felt relaxed. The total score ranged from 18 to 56 with a mean score of 36.0 ± 8.4 out of 60 (60%) (Table 2).

**Table 2:** Anxiety associated with MRI examination among the general population of the western region of Saudi Arabia MRI: magnetic resonance imaging, MRI-AQ: Magnetic Resonance Imaging-Anxiety Questionnaire, SD: standard deviation

MRI-AQ items	Not at all		Minimally		Moderately		Extremely	
	Number	%	Number	%	Number	%	Number	%
Anxiety symptoms								
I felt that I controlled the situation.	80	17.2%	168	36.1%	118	25.4%	99	21.3%
I had palpitations.	168	36.1%	183	39.4%	78	16.8%	36	7.7%
I found it hard to breathe.	242	52%	139	29.9%	54	11.6%	30	6.5%
I was afraid.	127	27.3%	170	36.6%	86	18.5%	82	17.6%
I wanted to come out.	155	33.3%	155	33.3%	59	12.7%	96	20.6%
I panicked.	228	49%	126	27.1%	51	11%	60	12.9%
I worried in advance.	92	19.8%	180	38.7%	95	20.4%	98	21.1%
I had to force myself to manage the situation.	95	20.4%	153	32.9%	86	18.5%	131	28.2%
Self-control was required when going through the examination.	128	27.5%	142	30.5%	84	18.1%	111	23.9%
I needed support and encouragement.	202	43.4%	116	24.9%	65	14%	82	17.6%
I wished to have someone with me.	215	46.2%	86	18.5%	47	10.1%	117	25.2%
I needed more detailed information.	121	26%	134	28.8%	81	17.4%	129	27.7%
Relaxation symptoms								
I felt relaxed.	236	50.8%	140	30.1%	64	13.8%	25	5.4%
I felt safe.	198	42.6%	162	34.8%	62	13.3%	43	9.2%
I felt calm.	201	43.2%	152	32.7%	58	12.5%	54	11.6%
Overall score								
Range	18-56							
Mean ± SD	36 ± 8.4							

**Figure 1:**
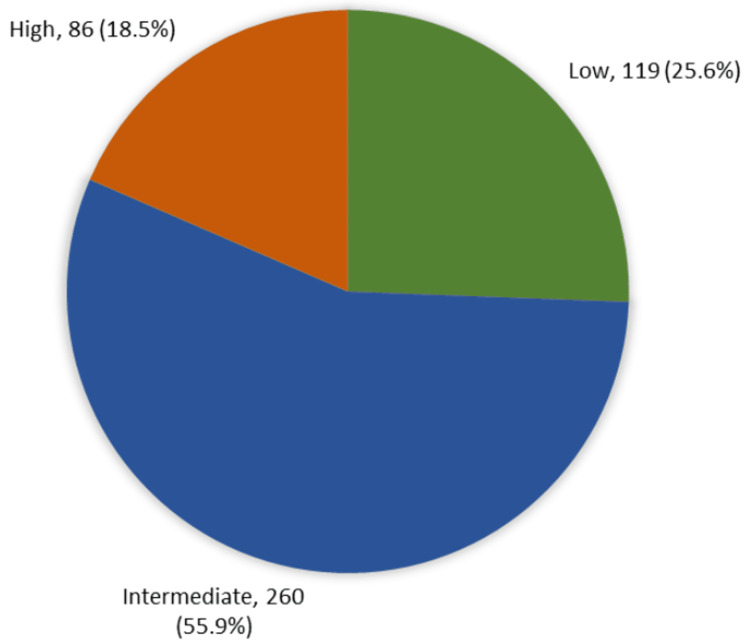
Overall anxiety level associated with MRI examination among the general population of the western region in Saudi Arabia MRI: magnetic resonance imaging

A total of 86 (18.5%) experienced high MRI-associated anxiety, 119 (25.6%) experienced low anxiety, and the vast majority of participants (55.9%, 260) experienced intermediate MRI-associated anxiety (Figure 1).

Just under one-quarter (23.6%) of the participants aged above 40 years had a high anxiety level compared with 11.1% of those aged 18-20 years (P = 0.113). For sex, 22.2% of the female participants showed high anxiety in comparison with 10.7% of the male participants (P = 0.001) (Table 3).

**Table 3:** Relation between MRI-associated anxiety and study participants’ age and gender MRI: magnetic resonance imaging, SD: standard deviation

Personal data	Anxiety level						P value	Mean ± SD
	Low (15-29)		Intermediate (30-44)		High (45-60)			
	Number	%	Number	%	Number	%		
Age in years							0.113	
18-20	14	19.4%	50	69.4%	8	11.1%		35.7 ± 6.8
21-30	53	29.9%	92	52%	32	18.1%		35.4 ± 8.6
31-40	17	23.6%	43	59.7%	12	16.7%		36.4 ± 8.3
>40	35	24.3%	75	52.1%	34	23.6%		36.7 ± 8.9
Gender							0.001	
Male	57	38.3%	76	51%	16	10.7%		33.4 ± 7.8
Female	62	19.6%	184	58.2%	70	22.2%		37.2 ± 8.4

## Discussion

As reported by a previous study, anxiety can affect the quality of an MRI examination because of the increased movement of patients during an examination [5]. Hence, we conducted this cross-sectional study to assess MRI examination-associated anxiety among the general population of the western region of Saudi Arabia. We found that 55.9% of the participants experienced intermediate anxiety during MRI screening, which is consistent with a similar study [4]. Concurring with these findings, another review study found that 37% of patients who underwent an MRI examination showed moderate to high levels of anxiety [5]. Moreover, claustrophobia, a type of anxiety disorder, was reported among 1%-15% of the patients, while 2.3% required sedation or were unable to proceed with the procedure [5].

Taking into account the difference between the number of female and male respondents, more female participants experienced intermediate to high levels of anxiety than male participants, which is similar to a published study [11]. However, another study found a higher degree of anxiety among male participants than female participants [4].

A previous study found that providing detailed information and instructions about the procedure lowered the degree of anxiety among patients [4]. Furthermore, poor knowledge of MRI among patients was reported in a recent study [12]. Therefore, since 73.9% of our participants stated a need for more precise information, we highly recommend providing adequate information, and appropriate answers and instructions for patients to help them control their fear and anxiety throughout the procedure.

Besides providing patients with a comprehensive explanation of the procedure, foot massage has been reported as an effective intervention to decrease anxiety before an MRI [11]. For this reason, further studies investigating the effectiveness of numerous interventions to prevent high levels of anxiety during MRI procedures are strongly suggested.

According to our findings, 43.9% of the study population had undergone an MRI examination, whereas 20% of MRI examinations have been found to be disrupted by significant motion secondary to anxiety [5]. Hence, there is a need for further studies investigating anxiety and its associated factors during MRI examination.

Limitations

Recall bias is possible since this was an online self-reported cross-sectional study to assess anxiety symptoms during an MRI examination. Moreover, an accurate method to exclude patients with a previous history of certain types of anxiety disorders should be used to limit possible bias that could affect the study results. There was also a significant difference between the number of female and male participants, which could affect the generalizability of the study’s results.

## Conclusions

More than half of our participants had intermediate anxiety associated with MRI examination. The majority were concerned beforehand and required more information in detail. Nearly half of the participants panicked and had trouble breathing. More female participants displayed high levels of anxiety compared with male participants, which was statistically significant. We highly recommend providing patients with sufficient information, adequate guidance, and answers that enable them to manage their anxiety and concerns throughout the MRI procedure.
